# Prealbumin as a prognostic indicator for hospital readmission of ulcerative colitis patients

**DOI:** 10.1093/pcmedi/pbad026

**Published:** 2023-11-10

**Authors:** Chao Ye, Anmin Wang, Wei Li, Wenyuan Li, Qi Shen, Zhangfei Wang, Li Xie, Qiuxia Jiang, Kaiguang Zhang, Shu Zhu

**Affiliations:** Department of Digestive Disease, The First Affiliated Hospital of University of Science and Technology of China (USTC), Division of Life Sciences and Medicine, University of Science and Technology of China, Hefei 230000, China; Department of Digestive Disease, The First Affiliated Hospital of University of Science and Technology of China (USTC), Division of Life Sciences and Medicine, University of Science and Technology of China, Hefei 230000, China; Institute of Immunology, the Chinese Academy of Sciences Key Laboratory of Innate Immunity and Chronic Disease, Division of Life Sciences and Medicine, University of Science and Technology of China (USTC), Hefei 230000, China; Department of Digestive Disease, The First Affiliated Hospital of University of Science and Technology of China (USTC), Division of Life Sciences and Medicine, University of Science and Technology of China, Hefei 230000, China; Department of Infectious Diseases, The First Affiliated Hospital of University of Science and Technology of China (USTC), Division of Life Sciences and Medicine, University of Science and Technology of China, Hefei 230000, China; Department of Radiotherapy, The First Affiliated Hospital of University of Science and Technology of China (USTC), Division of Life Sciences and Medicine, University of Science and Technology of China, Hefei 230000, China; Clinical laboratory, The First Affiliated Hospital of University of Science and Technology of China (USTC), Division of Life Sciences and Medicine, University of Science and Technology of China, Hefei 230000, China; Department of Digestive Disease, The First Affiliated Hospital of University of Science and Technology of China (USTC), Division of Life Sciences and Medicine, University of Science and Technology of China, Hefei 230000, China; Department of Digestive Disease, The First Affiliated Hospital of University of Science and Technology of China (USTC), Division of Life Sciences and Medicine, University of Science and Technology of China, Hefei 230000, China; Department of Digestive Disease, The First Affiliated Hospital of University of Science and Technology of China (USTC), Division of Life Sciences and Medicine, University of Science and Technology of China, Hefei 230000, China; Department of Digestive Disease, The First Affiliated Hospital of University of Science and Technology of China (USTC), Division of Life Sciences and Medicine, University of Science and Technology of China, Hefei 230000, China; Institute of Immunology, the Chinese Academy of Sciences Key Laboratory of Innate Immunity and Chronic Disease, Division of Life Sciences and Medicine, University of Science and Technology of China (USTC), Hefei 230000, China; Institute of Health and Medicine, Hefei Comprehensive National Science Center, Hefei 230000, China; School of Data Science, University of Science and Technology of China (USTC), Hefei 230000, China

Dear Editor,

Inflammatory bowel diseases (IBD) such as ulcerative colitis (UC) and Crohn's disease are affecting increasing numbers of patients worldwide.^1^ These chronic diseases significantly impact patients' quality of life as they are incurable and disabling.^1^ Patients with IBD have had an increasing rate of hospitalizations, half of all UC patients are hospitalized at least once during their lifetime, and readmissions after a UC-related hospitalization are common.^2^ Decreasing readmissions is important to healthcare systems, given the financial disincentive, and to patients to avoid the direct and indirect burden and costs of hospitalizations.^3^ Albumin is the most objective and applicable nutritional indicator.^4^ As a precursor for synthesizing albumin, prealbumin is barely influenced by external supplementation.^5^ Prealbumin can assist in estimating the nutritional state of a patient with more accuracy since it has a shorter average life than albumin.^5^ In addition, prealbumin was demonstrated to be an acute-phase reactant, and its concentration was altered during acute or chronic inflammatory states.^6^ As an important marker of nutritional and inflammatory state, serum prealbumin is used to evaluate the outcomes of several diseases, including heart failure^5^ and liver transplantation.^4^ Malnutrition and inflammation are common in patients with IBD.^1,2^ Thus, prealbumin may be valuable for nutritional and inflammatory screening and complement the assessment of severity and outcomes in IBD patients. Owing to a lack of studies on the association between prealbumin levels and UC, we conducted the present study to determine whether serum prealbumin levels in UC patients are associated with disease severity and hospital readmissions.

We conducted a prospective observational study of patients hospitalized in the First Affiliated Hospital of the University of Science and Technology of China. A total of 567 patients admitted due to active UC were included in the study. Informed consents have been obtained from the patients. All the patients discharged were registered. Patients were followed for 180 days to determine the rate of readmission (Fig. 1A). The baseline characteristics are presented in supplementary Table 1 (see online supplementary material), and the mean serum prealbumin of the total cohort was 19.26 ± 6.02 mg/dl. Patients with prealbumin ≤ 15 mg/dl and those with higher prealbumin are compared in supplementary Table 1. Patients with lower prealbumin were mostly women and patients with first-onset UC. Compared to patients with prealbumin > 15 mg/dl, those with prealbumin ≤ 15 mg/dl had lower hemoglobin, albumin, and total protein (TP), higher erythrocyte sedimentation rate (ESR), C-reactive protein (CRP), and platelet (PLT), and longer hospital stay (Fig. 1B). CRP and ESR were comprehensive markers of inflammation.^7^ CRP is an acute-phase reactant and a frequently used marker for assessing systemic inflammation. In contrast, prealbumin is a negative acute-phase protein since its synthesis is suppressed in the acute inflammatory phase.^4^ There were no differences in age, histories of smoking and drinking, and body mass index between the two groups (supplementary Table 1). Opportunistic infections are associated with nutritional status,^8^ we also found that patients in the low prealbumin group had higher rates of cytomegalovirus (CMV) (39.8% versus 8.6%, *P* < 0.001) and Epstein–Barr virus (EBV) (24.8% versus 3.0%, *P* < 0.001) infections. There were no differences in infection with fungus, *Mycobacterium tuberculosis*, and *Clostridium difficile* between the two groups (supplementary Table 1). Based on Truelove and Witts criteria and full colonoscopy examination, UC patients with prealbumin ≤ 15 mg/dl had more severe disease staging (*P* < 0.001) and more extensive (*P* < 0.001) and active (P < 0.001) endoscopic presentation. Additionally, patients with more severe colitis had lower prealbumin levels (Fig. 1C).

**Figure 1.**
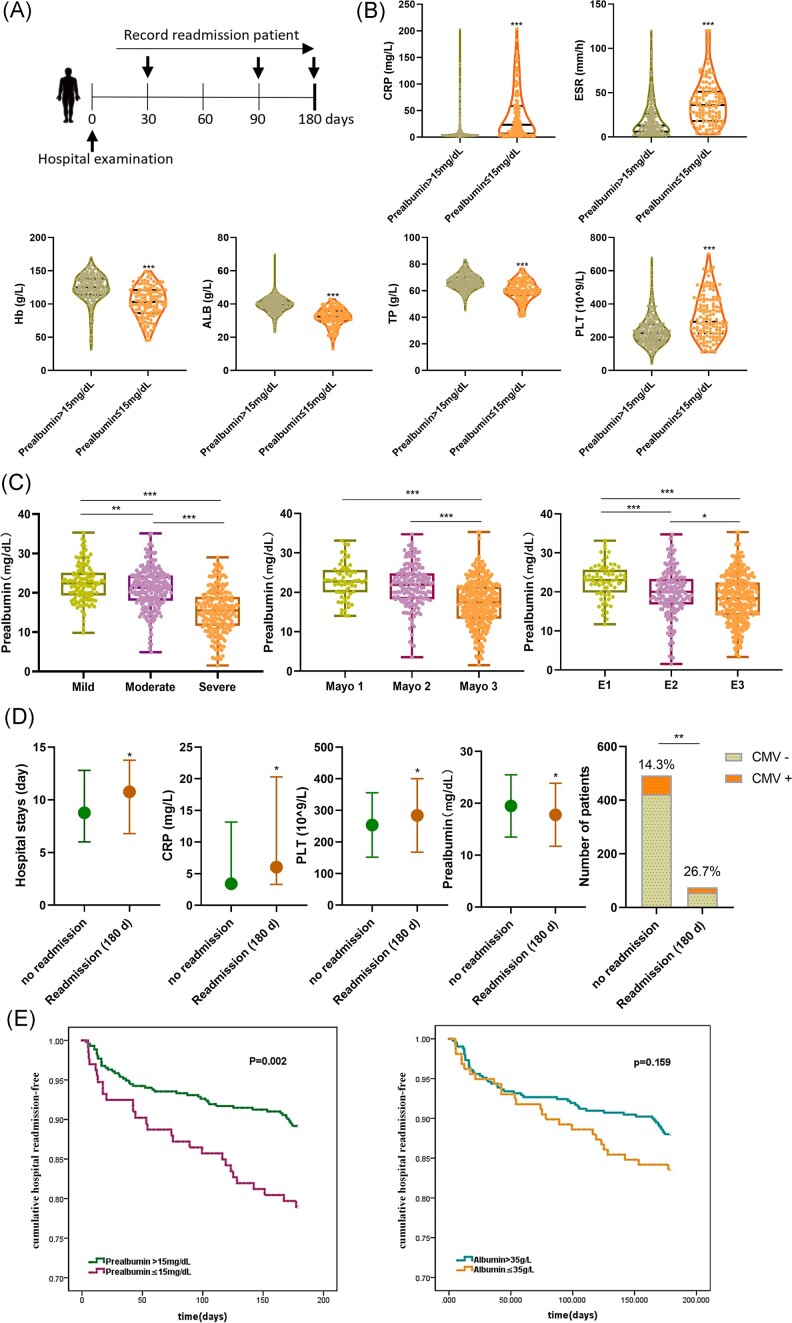
Low prealbumin level as an independent predictive factor for 180-day readmission in patients afflicted by UC. (**A**) Schematic diagram of experiment. (**B**) Patients with prealbumin ≤ 15 mg/dl had higher CRP, ESR, and PLT levels and lower Hb (Hemoglobin), ALB (Albumin), and TP (Total Protein) levels. Non-normally distributed continuous parameters were analyzed using the Mann–Whitney U test, ***P < 0.001. (**C**) Assessment of disease severity in UC patients using the Truelove and Witts criteria revealed that patients with severe disease exhibited lower levels of prealbumin compared to those with moderate and mild disease. Mayo endoscopic subscore, the severity of intestinal inflammation observed through endoscopy, was assessed in UC patients. Among the different Mayo subscore groups, patients with the most severe colitis (Mayo 3) exhibited lower levels of prealbumin compared to patients with moderate colitis (Mayo 2) and mild colitis (Mayo 1). Patients with pancolitis (E3) had lower levels of prealbumin compared to patients with left-sided colitis (E2) and proctitis (E1). ANOVA (Analysis of variance) analysis was conducted for quantitative data with multiple groups, *P  < 0.05, **P < 0.01, ***P < 0.001. (**D**) UC patients readmitted within 180 days had longer hospital stays, higher levels of CRP and PLT, and lower levels of prealbumin compared to patients who were not readmitted. In addition, they also had a higher incidence of CMV infection. Continuous parameters were analyzed by t-test (PLT and prealbumin) or Mann–Whitney U test (CRP) if data was nonnormally distributed. Categorical variables were analyzed using the chi-square test (CMV infection), *P  < 0.05, **P  < 0.01. (**E**) Kaplan–Meier curve of 180-day readmission according to low prealbumin (≤15 mg/dl) and normal prealbumin (>15 mg/dl). Patients with low prealbumin had significantly more readmissions than those with normal prealbumin (*P*  = 0.002) (left), while albumin exhibited only a non-significant trend (right).

Clinically, readmissions are common after hospitalization for UC and several risk factors have been associated with readmission after UC hospitalization. The most common reasons for readmission are disease flare, infections, and abdominal pain.^3^ Short-term readmissions represent a significant burden to the healthcare system.^3^ In our study, after 180 days of follow-up, 28 patients were readmitted within 30 days, 47 were readmitted within 90 days, and 75 were readmitted within 180 days. The 180-day readmission rate was 13.2%. Patients with lower prealbumin (prealbumin ≤ 15.0 mg/dl) had a higher rate of short-term (30-day, *P* = 0.043) and long-term (90-day, *P* = 0.012 and 180-day, *P* = 0.002) readmission (supplementary Table 2, see online supplementary material). Higher CRP, PLT, prealbumin levels, CMV infection rate, and longer duration of hospital stay were also associated with the 180-day readmission (Fig. 1D). In the multivariate logistic regression to identify the independent predictors of rehospitalization at 180 days, low prealbumin levels were significantly associated with 180-day readmission (odds ratio: 2.029, 95% confidence interval: 1.076–3.892, *P* = 0.029) (supplementary Table 3, see online supplementary material). Figure 1E shows the Kaplan–Meier curves for 180-day readmission by prealbumin levels (below and above 15 mg/dl). Patients with low prealbumin had significantly more readmissions than those with normal prealbumin (*P* = 0.002). It is worth noting that while previously reported potential UC risk factors such as admission albumin^9^ exhibited only a non-significant trend, prealbumin demonstrated a more robust association with readmission, thus underscoring prealbumin's superior predictive utility.

In conclusion, our study revealed a link between low prealbumin levels (≤15 mg/dl) at admission and elevated readmission risks in patients with active UC, signifying its potential as an important predictor. This insight could aid significantly in devising targeted interventions, thereby alleviating healthcare burden and reducing readmission rates.

## Supplementary Material

pbad026_Supplemental_File

## References

[1] Molodecky NA, Soon IS, Rabi DM, et al. Increasing incidence and prevalence of the inflammatory bowel diseases with time, based on systematic review. Gastroenterology. 2012;142(1):46–54. doi:10.1053/j.gastro.2011.10.001. 22001864

[2] Samuel S, Ingle SB, Dhillon S, et al. Cumulative incidence and risk factors for hospitalization and surgery in a population-based cohort of ulcerative colitis. Inflamm Bowel Dis. 2013;19(9):1858–66. doi:10.1097/MIB.0b013e31828c84c5. 23660997 PMC4526131

[3] Mudireddy P, Scott F, Feathers A, Lichtenstein GR. Inflammatory bowel disease: Predictors and causes of early and late hospital readmissions. Inflamm Bowel Dis. 2017;23(10):1832–9. doi:10.1097/MIB.0000000000001242. 28858068

[4] Li Y, Liu X, Jiang Y, et al. Low preoperative prealbumin predicts the prevalence of complications following liver transplantation. BMC Gastroenterol. 2021;21(1):233. doi:10.1186/s12876-021-01818-1. 34022800 PMC8141182

[5] Lourenço P, Silva S, Friões F, et al. Low prealbumin is strongly associated with adverse outcome in heart failure. Heart. 2014;100(22):1780–5. doi:10.1136/heartjnl-2014-305747. 24986895

[6] Ni M, Wei W, Feng Q, et al. Transthyretin as a potential serological marker for the diagnosis of patients with early rheumatoid arthritis. Clin Exp Rheumatol. 2013;31(3):394–9. 23465306

[7] Lapić I, Padoan A, Bozzato D, et al., Erythrocyte sedimentation rate and C-reactive protein in acute inflammation. Am J Clin Pathol. 2020;153(1):14–29. doi:10.1093/ajcp/aqz142. 31598629

[8] Katona P, Katona‐Apte J. The interaction between nutrition and infection. Clin Infect Dis. 2008;46(10):1582–8. doi:10.1086/587658. 18419494

[9] Tinsley A, Naymagon S, Mathers B, et al. Early readmission in patients hospitalized for ulcerative colitis: incidence and risk factors. Scand J Gastroenterol. 2015;50(9):1103–9. doi:10.3109/00365521.2015.1020862. 25866237

