# Supplementary material for: Prealbumin as a prognostic indicator for hospital readmission of ulcerative colitis patients
Source: Precis Clin Med. 2023 Nov 10;7(1):pbad026. doi: 10.1093/pcmedi/pbad026 (PMC10773210; doi:10.1093/pcmedi/pbad026)
Supplement: pbad026_Supplemental_File [file pbad026_supplemental_file.docx]

**Supplementary Materials**

**Table 1. Characteristics of patients and comparison between those with prealbumin ≤15 mg/dL and >15 mg/dL**

|  | **Total** | **Prealbumin**  **≤15** **mg/dL** | **Prealbumin**  **>15 mg/dL** | **p** |
| --- | --- | --- | --- | --- |
| **Number of patients** | 567 | 133 | 434 |  |
| Age (years) | 46.1 ± 15.3 | 47.1 ± 16.4 | 45.8 ± 14.9 | 0.408 |
| male/female (n, %) | 324/243 (57.1/42.9) | 62/71 (46.6/53.4) | 262/172 (60.4/39.6) | **0.005** |
| Smoking (n, %) | 49 (8.6) | 13 (9.8) | 36 (8.3) | 0.595 |
| Drinking (n, %) | 47 (8.3) | 7 (5.3) | 40 (9.2) | 0.148 |
| BMI | 21.7 ± 3.3 | 21.0 ± 3.6 | 21.9 ± 3.2 | 0.082 |
| [Initial onset  /Chronic recurrence (n, %)] | 150/417 (26.5/73.5) | 46/87  (34.6/65.4) | 104/330 (24.0/76.0) | **0.015** |
| Hospital stay (day) | 8.8 (6.7–12.8) | 12.7 (7.9–19.7) | 7.8 (6.7–12.8) | **<0.001** |
| **Opportunistic infection (n, %)** |  |  |  |  |
| CMV | 90 (16.0) | 53 (39.8) | 37 (8.6) | **<0.001** |
| EBV | 46 (8.1) | 33 (24.8) | 13 (3.0) | **<0.001** |
| Fungal | 78 (13.8) | 23 (17.3) | 55 (12.7) | 0.176 |
| Mycobacterium Tuberculosis | 56 (9.9) | 17 (12.8) | 39 (9.0) | 0.199 |
| **Disease severity (n, %)** |  |  |  | **<0.001** |
| Mild | 119 (21.0) | 4 (3.0) | 115 (26.5) |  |
| Moderate | 239 (42.2) | 30 (22.6) | 209 (48.2) |  |
| Severe | 209 (36.9) | 99 (74.4) | 110 (25.3) |  |
| **Disease extent (n, %)** |  |  |  | **<0.001** |
| E1 (proctitis) | 70 (12.3) | 4 (3.0) | 66 (15.2) |  |
| E2 (left-sided colitis) | 184 (32.5) | 30 (22.6) | 154 (35.5) |  |
| E3 (pancolitis) | 313 (55.2) | 99 (74.4) | 214 (49.3) |  |
| **Endoscopic activities (n, %)** |  |  |  | **<0.001** |
| Mayo 1 | 64 (11.3) | 3 (2.3) | 61 (14.1) |  |
| Mayo 2 | 177 (31.2) | 19 (14.3) | 158 (36.4) |  |
| Mayo 3 | 326 (57.5) | 111 (83.5) | 215 (49.5) |  |

**Table 2. Univariate analysis for 30-day, 90-day, and 180-day readmission of patients with active UC**

|  | **30-day**  **Readmission**  **/Not** | **p** | **90-day**  **Readmission**  **/Not** | **p** | **180-day**  **Readmission**  **/Not** | **p** |
| --- | --- | --- | --- | --- | --- | --- |
| **Number of patients** | 28/539 |  | 47/520 |  | 75/492 |  |
| Age (years) | 45.3 ± 16.4  /46.1 ±15.2 | 0.782 | 43.8 ± 17.0  /46.3 ± 15.1 | 0.288 | 43.7 ± 16.5  /46.5 ± 15.1 | 0.145 |
| Male gender (n, %) | 16/308  (57.1/57.1) | 1.000 | 29/295  (61.7/56.7) | 0.510 | 45/279  (60/56.7) | 0.591 |
| Smoking (n, %) | 2/47  (7.1/8.7) | 1.000^#^ | 6/43  (12.8/8.3) | 0.436^#^ | 7/42  (9.3/8.5) | 0.819 |
| Drinking (n, %) | 1/46  (3.6/8.5) | 0.564^#^ | 2/45  (4.3/8.7) | 0.441^#^ | 6/41  (8.0/8.3) | 0.922 |
| BMI | 25.1 ± 4.7  /21.6 ± 3.3 | 0.011 | 23.6 ± 4.6  /21.6 ± 3.3 | 0.072 | 22.7 ± 4.0  /21.6 ± 3.3 | 0.156 |
| Hospital stay (day) | 13.3 ± 11.2  /10.7 ± 6.6 | 0.232^#^ | 12.7 ± 9.2  /10.6 ± 6.6 | 0.054 | 10.8 (6.8-13.8)/8.8 (6.0-13.7) | **0.019** |
| Initial onset (n, %) | 5/145  (17.9/26.9) | 0.290 | 12/138  (25.5/26.5) | 0.881 | 21/129  (28.0/26.2) | 0.745 |
| **Laboratory tests** |  |  |  |  |  |  |
| CRP (mg/L) | 27.2 ± 36.9  /17.6 ± 31.6 | 0.131 | 6.4(3.5-14.0)  /3.6(3.1-13.9) | 0.030 | 6.0 (3.3-20.3)  /3.4 (3.1-13.2) | **0.038** |
| ESR (mm/h) | 31.3 ± 27.3  /23.8 ± 22.4 | 0.099 | 28.3 ± 23.7  /23.7 ± 22.6 | 0.204 | 27.6 ± 23.5  /23.6 ± 22.6 | 0.167 |
| HB (g/L) | 119.3 ± 25.5  /118.8 ± 22.8 | 0.905 | 117.7 ± 24.8  /118.9 ± 22.7 | 0.731 | 116.6 ± 25.1  /119.1 ± 22.5 | 0.373 |
| PLT (10^9/L) | 270.9 ± 118.7  /255.2 ± 102 | 0.432 | 276.0 ± 109.1  /254.1 ± 102.2 | 0.163 | 279.2 ± 113.9  /252.4 ± 100.7 | **0.036** |
| ALB (g/L) | 37.5 ± 4.9  /37.5 ± 5.6 | 0.997 | 36.5 ± 6.6  /37.6 ± 5.5 | 0.192 | 37.0 ± 6.0  /37.6 ± 5.6 | 0.363 |
| TP (g/L) | 66.4 ± 7.5  /64.9 ± 6.7 | 0.255 | 64.5 ± 8.0  /65.0 ± 6.7 | 0.645^$^ | 65.0 ± 7.1  /65.0 ± 6.7 | 0.953 |
| **Opportunistic infection (n, %)** |  |  |  |  |  |  |
| CMV | 8/82  (28.6/15.2) | 0.109^#^ | 14/76  (29.8/14.7) | 0.007 | 20/70  (26.7/14.3) | **0.007** |
| EBV | 3/43  (10.7/8.0) | 0.871^#^ | 5/41  (10.6/7.9) | 0.702^#^ | 6/40  (8.0/8.1) | 0.969^#^ |
| Fungal | 4/74  (14.3/13.7) | 1.000^#^ | 9/69  (19.1/13.3) | 0.262 | 14/64  (18.7/13.0) | 0.185 |
| Mycobacterium Tuberculosis | 7/49  (25.0/9.1) | 0.015^#^ | 9/47  (19.1/9.0) | 0.049^#^ | 10/46  (13.3/9.3) | 0.281 |
| **Disease severity (n, %)** |  | 0.560 |  | 0.967 |  | 0.520 |
| Mild | 5/114  (17.9/21.2) |  | 10/109  (21.3/21.0) |  | 12/107  (16.0/21.7) |  |
| Moderate | 10/229  (35.7/42.5) |  | 19/220  (40.4/42.3) |  | 34/205  (45.3/41.7) |  |
| Severe | 13/196  (46.4/36.4) |  | 18/191  (38.3/36.7) |  | 29/180  (38.7/36.6) |  |
| **Disease extent (n, %)** |  | 0.250^##^ |  | 0.645 |  | 0.166 |
| E1 (proctitis) | 1/69  (3.6/12.8) |  | 5/65  (10.6/12.5) |  | 7/63  (9.3/12.8) |  |
| E2 (left-sided colitis) | 10/174  (35.7/32.3) |  | 13/171  (27.7/32.9) |  | 19/165  (25.3/33.5) |  |
| E3 (pancolitis) | 17/296  (60.7/54.9) |  | 29/284  (61.7/54.6) |  | 49/264  (65.3/53.7) |  |
| **Endoscopic activities (****n, %)** |  | 0.991^##^ |  | 0.955 |  | 0.615 |
| Mayo 1 | 3/61  (10.7/11.3) |  | 5/59  (10.6/11.3) |  | 6/58  (8.0/11.8) |  |
| Mayo 2 | 9/168  (32.1/31.2) |  | 14/163  (29.8/31.3) |  | 25/152  (33.3/30.9) |  |
| Mayo 3 | 16/310  (57.1/57.5) |  | 28/298  (59.6/57.3) |  | 44/282  (58.7/57.3) |  |
| Mean level of Prealbumin (mg/dL) | 17.56±5.36  /19.35±6.5 | 0.126 | 17.77±6.12  /19.40±6.0 | 0.076 | 17.80±6.08/19.49±5.99 | **0.024** |
| **Prealbumin≤15 mg/dL (n, %)** | 11/122  (39.3/22.6) | **0.043** | 18/115  (38.3/22.1) | **0.012** | 28/105  (37.3/21.3) | **0.002** |

^#^: Yates correction for continuity; ^##^: likelihood ratio test; ^$^: unequal variances *t*-test

**Table 3. Multivariate analysis for 180-day hospital readmission**

| **Variable** | **Odds ratio** | **95% CI** | **p** |
| --- | --- | --- | --- |
| CRP >10 (mg/L) | 1.146 | 0.459–1.661 | 0.678 |
| PLT >300 (10^9^/L) | 1.016 | 0.547–1.886 | 0.960 |
| Hospital stays | 1.011 | 0.975–1.049 | 0.554 |
| CMV infection | 1.732 | 0.904–3.315 | 0.098 |
| Prealbumin ≤15(mg/dL) | 2.029 | 1.076–3.892 | **0.029** |

**Patients and Study Design**

We conducted a prospective observational study of patients hospitalized in the First Affiliated Hospital of the University of Science and Technology of China between January 2015 and December 2021. Patients with a primary diagnosis of active UC were eligible for the study. UC was diagnosed according to current international guidelines on the diagnosis and treatment of UC. Active UC was identified as UC with purulent hematochezia and diarrhea more than three times per day. All included patients underwent full colonoscopy during hospitalization and were followed up for 180 days. Patients with no admission prealbumin, no endoscopy examination, incomplete data, or who were lost to follow-up were excluded. All the patients discharged were registered. Patients were followed for 180 days to determine the rate of readmission. In total, 567 patients were included in the cohort. All patients received excellent care during the hospitalization.

**Demographic and Clinical Variables**

Demographic and clinical variables, including gender, age, smoking and drinking history, body mass index (BMI), length of hospital stay, disease type (initial onset or chronic recurrence), disease severity, and endoscopy activity, were reported in this study. Disease severity was evaluated using the Truelove and Witts Criteria. The endoscopic activity was assessed using Mayo endoscopic subscore. The details of Mayo endoscopic subscore were as follows: 0 = normal or inactive disease; 1 = erythema, decreased vascular pattern; 2 = marked erythema, absent vascular pattern, any friability, and erosions; and 3 = spontaneous bleeding, ulceration. The location and extent of the disease were evaluated using the Montreal classification: proctitis (E1), left-sided colitis (E2), or pancolitis (E3) was determined as previously reported.

**Laboratory Testing**

Fasting peripheral venous blood samples were collected within 24 h of admission to measure prealbumin levels. Laboratory parameters were also assessed, including erythrocyte sedimentation rate (ESR), C-reactive protein (CRP), hemoglobin, platelet, albumin, and total protein (TP). The quantitative determination of Cytomegalovirus (CMV) and Epstein-Barr virus (EBV) DNA was performed by real-time quantitative Polymerase Chain Reaction (qPCR) on peripheral blood using the Human CMV nucleic acid quantitative detection kit (DAAN GENE, Guangzhou, China) and Human EBV nucleic acid quantitative detection kit (DAAN GENE, Guangzhou, China). CMV and BV infection was defined as positive qPCR levels. We used T-Spot. *TB* (Immune Spot Method) on peripheral blood to detect *Mycobacterium tuberculosis* infection. The feces of patients were also collected and tested for *Clostridium difficile* and fungus.

**Ethical statement**

The anonymity of patients enrolled in the study was carefully protected, and investigations reported in the manuscript were performed with written informed consent. This study was conducted in accordance with the Declaration of Helsinki and approved by the Ethics Committee of the coordinating Centre (the First Affiliated Hospital of the University of Science and Technology of China).

**Statistical Analysis**

Data were analyzed using SPSS 22.0 (IBM Corp., Armonk, New York, USA). Categorical variables were expressed as numbers or percentages, and continuous variables were expressed as means with standard deviation or medians with interquartile range (IQR). Patients divided into two groups according to the prealbumin values were compared. Risk factors of readmissions within 30, 90, and 180 days were also analyzed. Non-normally distributed continuous variables were analyzed using the independent t-test or Mann-Whitney U test if data was nonnormal distribution. ANOVA analysis was conducted for quantitative data with multiple groups. Categorical variables were analyzed using the Chi-square test as appropriate. Variables considered as significant predictors by univariate analysis were used for multivariate logistic regression analysis to identify independent predictive factors of 180 days readmission. And by means of the Kaplan-Meier method the curves of accumulated events were performed to observe the prognostic differences between the patients with prealbumin ≤15.0 mg/dL and those with prealbumin >15 mg/dL. The two-sided p value less than 0.05 was considered statistically significant.
